# Supplementary material for: A UMLS-based spell checker for natural language processing in vaccine safety
Source: BMC Med Inform Decis Mak. 2007 Feb 12;7:3. doi: 10.1186/1472-6947-7-3 (PMC1805499; doi:10.1186/1472-6947-7-3)
Supplement: Additional file 13 — RAP application directory. Sets up directory for RAP (RDF application for PHP) source code [file 1472-6947-7-3-S13.gz › rap/doc/phpdoc/default/FOAF_RES.html]

xml version="1.0" encoding="iso-8859-1"?


Docs For Class FOAF\_RES


## Class FOAF\_RES

Description

Description |
Methods (details)

Located in /vocabulary/FOAF\_RES.php (line 22)

Method Summary

Description |
Methods (details)

void
ACCOUNT\_NAME
()

void
ACCOUNT\_SERVICE\_HOMEPAGE
()

void
AGENT
()

void
AIM\_CHAT\_ID
()

void
BASED\_NEAR
()

void
CURRENT\_PROJECT
()

void
DEPICTION
()

void
DEPICTS
()

void
DNA\_CHECKSUM
()

void
DOCUMENT
()

void
FAMILY\_NAME
()

void
FIRST\_NAME
()

void
FUNDED\_BY
()

void
GEEKCODE
()

void
GENDER
()

void
GIVENNAME
()

void
GROUP
()

void
HOLDS\_ACCOUNT
()

void
HOMEPAGE
()

void
ICQ\_CHAT\_ID
()

void
IMAGE
()

void
IMG
()

void
INTEREST
()

void
JABBER\_ID
()

void
KNOWS
()

void
LOGO
()

void
MADE
()

void
MAKER
()

void
MBOX
()

void
MBOX\_SHA1SUM
()

void
MEMBER
()

void
MEMBERSHIP\_CLASS
()

void
MSN\_CHAT\_ID
()

void
MYERS\_BRIGGS
()

void
NAME
()

void
NICK
()

void
ONLINE\_ACCOUNT
()

void
ONLINE\_CHAT\_ACCOUNT
()

void
ONLINE\_ECOMMERCE\_ACCOUNT
()

void
ONLINE\_GAMING\_ACCOUNT
()

void
ORGANIZATION
()

void
PAGE
()

void
PAST\_PROJECT
()

void
PERSON
()

void
PERSONAL\_PROFILE\_DOCUMENT
()

void
PHONE
()

void
PLAN
()

void
PRIMARY\_TOPIC
()

void
PROJECT
()

void
PUBLICATIONS
()

void
SCHOOL\_HOMEPAGE
()

void
SHA1
()

void
SURNAME
()

void
THEME
()

void
THUMBNAIL
()

void
TIPJAR
()

void
TITLE
()

void
TOPIC
()

void
TOPIC\_INTEREST
()

void
WEBLOG
()

void
WORKPLACE\_HOMEPAGE
()

void
WORK\_INFO\_HOMEPAGE
()

void
YAHOO\_CHAT\_ID
()

Methods

Description |
Methods (details)

ACCOUNT\_NAME (line 96)

void

ACCOUNT\_NAME
()

ACCOUNT\_SERVICE\_HOMEPAGE (line 102)

void

ACCOUNT\_SERVICE\_HOMEPAGE
()

AGENT (line 24)

void

AGENT
()

AIM\_CHAT\_ID (line 108)

void

AIM\_CHAT\_ID
()

BASED\_NEAR (line 114)

void

BASED\_NEAR
()

CURRENT\_PROJECT (line 120)

void

CURRENT\_PROJECT
()

DEPICTION (line 126)

void

DEPICTION
()

DEPICTS (line 132)

void

DEPICTS
()

DNA\_CHECKSUM (line 138)

void

DNA\_CHECKSUM
()

DOCUMENT (line 30)

void

DOCUMENT
()

FAMILY\_NAME (line 144)

void

FAMILY\_NAME
()

FIRST\_NAME (line 150)

void

FIRST\_NAME
()

FUNDED\_BY (line 156)

void

FUNDED\_BY
()

GEEKCODE (line 162)

void

GEEKCODE
()

GENDER (line 168)

void

GENDER
()

GIVENNAME (line 174)

void

GIVENNAME
()

GROUP (line 36)

void

GROUP
()

HOLDS\_ACCOUNT (line 180)

void

HOLDS\_ACCOUNT
()

HOMEPAGE (line 186)

void

HOMEPAGE
()

ICQ\_CHAT\_ID (line 192)

void

ICQ\_CHAT\_ID
()

IMAGE (line 42)

void

IMAGE
()

IMG (line 198)

void

IMG
()

INTEREST (line 204)

void

INTEREST
()

JABBER\_ID (line 210)

void

JABBER\_ID
()

KNOWS (line 216)

void

KNOWS
()

LOGO (line 222)

void

LOGO
()

MADE (line 228)

void

MADE
()

MAKER (line 234)

void

MAKER
()

MBOX (line 240)

void

MBOX
()

MBOX\_SHA1SUM (line 246)

void

MBOX\_SHA1SUM
()

MEMBER (line 252)

void

MEMBER
()

MEMBERSHIP\_CLASS (line 258)

void

MEMBERSHIP\_CLASS
()

MSN\_CHAT\_ID (line 264)

void

MSN\_CHAT\_ID
()

MYERS\_BRIGGS (line 270)

void

MYERS\_BRIGGS
()

NAME (line 276)

void

NAME
()

NICK (line 282)

void

NICK
()

ONLINE\_ACCOUNT (line 48)

void

ONLINE\_ACCOUNT
()

ONLINE\_CHAT\_ACCOUNT (line 54)

void

ONLINE\_CHAT\_ACCOUNT
()

ONLINE\_ECOMMERCE\_ACCOUNT (line 60)

void

ONLINE\_ECOMMERCE\_ACCOUNT
()

ONLINE\_GAMING\_ACCOUNT (line 66)

void

ONLINE\_GAMING\_ACCOUNT
()

ORGANIZATION (line 72)

void

ORGANIZATION
()

PAGE (line 288)

void

PAGE
()

PAST\_PROJECT (line 294)

void

PAST\_PROJECT
()

PERSON (line 78)

void

PERSON
()

PERSONAL\_PROFILE\_DOCUMENT (line 84)

void

PERSONAL\_PROFILE\_DOCUMENT
()

PHONE (line 300)

void

PHONE
()

PLAN (line 306)

void

PLAN
()

PRIMARY\_TOPIC (line 312)

void

PRIMARY\_TOPIC
()

PROJECT (line 90)

void

PROJECT
()

PUBLICATIONS (line 318)

void

PUBLICATIONS
()

SCHOOL\_HOMEPAGE (line 324)

void

SCHOOL\_HOMEPAGE
()

SHA1 (line 330)

void

SHA1
()

SURNAME (line 336)

void

SURNAME
()

THEME (line 342)

void

THEME
()

THUMBNAIL (line 348)

void

THUMBNAIL
()

TIPJAR (line 354)

void

TIPJAR
()

TITLE (line 360)

void

TITLE
()

TOPIC (line 366)

void

TOPIC
()

TOPIC\_INTEREST (line 372)

void

TOPIC\_INTEREST
()

WEBLOG (line 378)

void

WEBLOG
()

WORKPLACE\_HOMEPAGE (line 390)

void

WORKPLACE\_HOMEPAGE
()

WORK\_INFO\_HOMEPAGE (line 384)

void

WORK\_INFO\_HOMEPAGE
()

YAHOO\_CHAT\_ID (line 396)

void

YAHOO\_CHAT\_ID
()

Documentation generated on Fri, 17 Dec 2004 16:14:57 +0100 by phpDocumentor 1.3.0RC3
